# Nutrient intake of pregnant women at high risk of gestational diabetes

**DOI:** 10.3402/fnr.v59.26676

**Published:** 2015-05-19

**Authors:** Jelena Meinilä, Saila B. Koivusalo, Anita Valkama, Kristiina Rönö, Maijaliisa Erkkola, Hannu Kautiainen, Beata Stach-Lempinen, Johan G. Eriksson

**Affiliations:** 1 Group Administration, Research and Development, The Hospital District of Helsinki and Uusimaa, Helsinki, Finland; 2 Department of Obstetrics and Gynecology, Helsinki University Central Hospital, Helsinki, Finland; 3 Folkhälsan Research Centre, Helsingfors Universitet, Helsinki, Finland; 4 Department of Obstetrics and Gynecology, University of Helsinki, Helsinki, Finland; 5 Department of Food and Environmental Sciences, University of Helsinki, Helsinki, Finland; 6 Department of Obstetrics and Gynecology, South-Karelia Central Hospital, Lappeenranta, Finland; 7 Department of General Practice and Primary Health Care, University of Helsinki, Helsinki, Finland

**Keywords:** nutrition, pregnancy, diabetes, obesity, maternal nutrition, diet

## Abstract

**Background:**

The prevalence of gestational diabetes (GDM) has been increasing along with the obesity pandemic. It is associated with pregnancy complications and a risk of type 2 diabetes.

**Objective:**

To study nutrient intake among pregnant Finnish women at increased risk of GDM due to obesity or a history of GDM.

**Design:**

Food records from obese women or women with GDM history (*n=*394) were examined at baseline (≤20 weeks of pregnancy) of the Finnish Gestational Diabetes Prevention Study.

**Results:**

The pregnant women had a mean fat intake of 33 en% (SD 7), saturated fatty acids (SFA) 12 en% (SD 3), and carbohydrate 46 en% (SD 6). Sucrose intake among pregnant women with GDM history was 7 en% (SD 3), which was different from the intake of the other pregnant women, 10 en% (SD 4) (*p<*0.001). Median intakes of folate and vitamins A and D provided by food sources were below the Finnish national nutrition recommendation, but, excluding vitamin A, supplements raised the total intake to the recommended level. The frequency of use of dietary supplements among pregnant women was 77%.

**Conclusions:**

The observed excessive intake of SFA and low intake of carbohydrates among women at high risk of GDM may further increase their risk of GDM. A GDM history, however, seems to reduce sucrose intake in a future pregnancy. Pregnant women at high risk of GDM seem to have insufficient intakes of vitamin D and folate from food and thus need supplementation, which most of them already take.

Along with the obesity pandemic, the prevalence of GDM has increased. In Finland, approximately one-third of pregnant women are overweight or obese (1), and the prevalence of GDM was 13% in 2012 (2, 3). GDM is the result of an interaction between genetic and environmental risk factors, including modifiable lifestyle factors such as a non-optimal nutrition and a sedentary lifestyle (4, 5). GDM complicates pregnancies and elevates the future risk of type 2 diabetes of the mother and offspring (6–11). Diets low in carbohydrates and high in total fat and SFA during pregnancy are associated with increased risk of GDM (12, 13), and a low-glycemic-index diet with decreased risk (14).

The Finnish nutrition recommendations (15) are based on the Nordic nutrition recommendations (16). Thus, the Nordic and Finnish recommendations are nearly the same for nutrient intakes. They acknowledge the dietary needs of overweight and obese pregnant women (15). The main objective of the recommendations for overweight and obese women is preventing excessive weight gain during pregnancy. Otherwise, the recommendations for dietary choices are similar to those recommended for all pregnant women. The Finnish recommendations for pregnant women emphasize the importance of adequate intake of folate, iron, essential fatty acids (EFA), and vitamin D from food sources. Supplemental intake of vitamin D (10 µg/day) is recommended, whereas no common recommendation for supplementation of iron, folic acid, and calcium is provided, as these require individual assessment. Delivering this information to pregnant women is one of the main tasks of Finnish maternity clinics, as part of the organized public health-care system in Finland.

To date, little knowledge exists about diet and nutrient intake of pregnant women at high risk of GDM due to obesity or a history of GDM. To make diet modifications, it is essential to know where the problem lies. In the present study, we examined and described the first-trimester nutrient intake of pregnant women at high risk of GDM. We examined the results and discussed the sufficiency of intakes in reference to the Finnish national nutrition recommendations (15). In addition, we studied the nutrient intake in relation to GDM history and considered the possible differences between the groups. The hypothesis was that receiving dietary advice during earlier pregnancies and a having a history of GDM would affect the nutrient intake in the current pregnancy. We report here the nutrients that are critical during pregnancy and those with an established association with GDM.

## Materials and methods

The Finnish Gestational Diabetes Prevention Study (RADIEL) is a randomized, controlled lifestyle intervention study that started in 2008 in the cities of Helsinki, Espoo, Vantaa, and Lappeenranta. Between February 2008 and November 2011, RADIEL recruited a total of 788 women, of whom 234 were eligible for the current study. Participants were at 20 weeks or less of gestation or planning a pregnancy; all were at elevated risk of GDM because of obesity (BMI≥30 kg/m^2^) or a history of GDM.

Exclusion criteria for RADIEL included age under 18 years, diabetes mellitus prior to pregnancy, medication influencing glucose homeostasis prior to enrolment, multiple pregnancy, physical disability, alcohol and drug abuse, severe psychiatric condition, and significant difficulty in communication (for example, absence of common language).

**Fig. 1:**
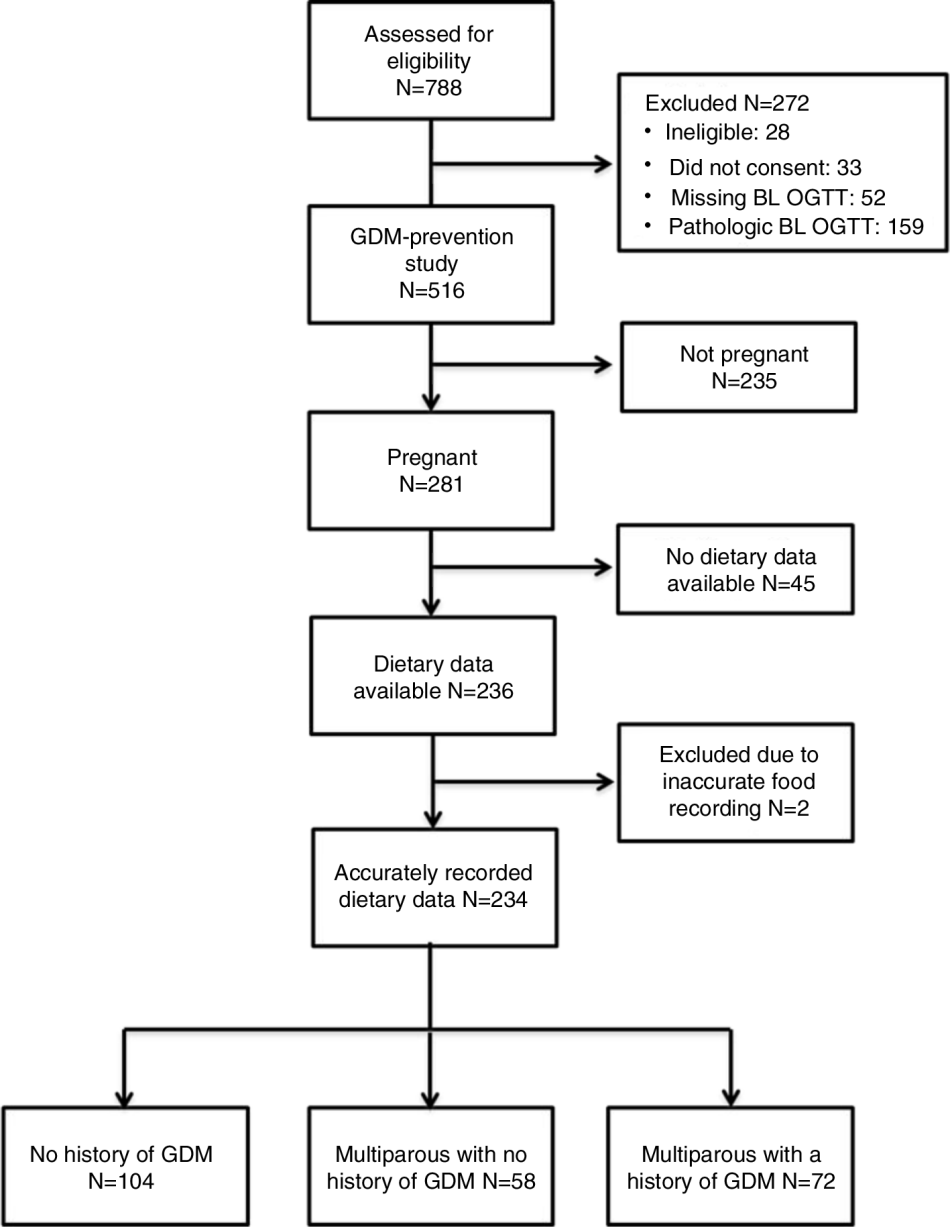
Selection of the subjects of the study. BL, baseline; OGTT, oral glucose tolerance test.

Pregnant women were recruited at primary health-care centers and antenatal clinics, as well as by newspaper advertisements and targeted social media announcements. Figure 1 shows the selection of the participants for the current study. The study design has been described in great detail previously (17). The current paper focuses on the nutrient intake of pregnant women at high risk of GDM at baseline participating in the RADIEL intervention study.

After enrolment, all subjects received a letter with instructions for completing a 3-day food record, which they were to return to a study nurse at a scheduled appointment. The subjects filled out a background questionnaire encompassing sociodemographics, earlier pregnancies and deliveries, and health-related behaviors during the previous 6 months. Information on GDM was based upon the subject's medical record and physician confirmation, except for two subjects for whom physician confirmation was unavailable. Prepregnancy BMI was calculated from height measured at the study visit and self-reported prepregnancy weight.

Instructions for the food records were to complete them over three consecutive days (two weekdays and a weekend day), using food and beverage labels as accurately as possible, and to report amounts used as household measures, such as a teaspoon, a glass, a scoop, or as weight if available. Vitamin and mineral supplements were recorded by their brand names and the amounts as tablets, drops, spoonfuls, or milliliters.

Two trained nutritionists assessed and entered the data from the food records into a nutritional calculation software program, AivoDiet, version 2.0.1.5 (Aivo Finland Oy, Turku, Finland) for computation of the nutrient intake. During this process, a table of usual portion sizes was used to help convert household measures and volumes to grams (18). The food composition database in the software was provided by the Finnish National Institute for Health and Welfare (www.fineli.fi). The analytical nutrient values in the database are mostly based on Finnish studies. In addition, complementary data were obtained from the Finnish food industry and international food composition tables. The national food composition database contains standard recipes that are based on those available in contemporary Finnish cookery books. If a food or recipe comparable to that in the diary was lacking, a new recipe was created based on the information given in the diary. A separate question about supplement type, label, and utilization frequency provided for computing an additional variable for a nutrient intake including the intake from both food and supplements. These data enabled calculation of the mean intakes of nutrients, which were then transferred into the statistical software Stata, version 13.1 (Stata Corporation, College Station, TX, USA). To assess the adequacy of nutrient intake, we used the national recommendations published in 2005 (19), which were valid during the period in which the food intake data was collected.

### Statistical analyses

The data are presented as means with standard deviations, medians with interquartile range, or counts with percentages. Statistical significance for the hypotheses was evaluated by using generalized linear models with appropriate distribution and link function. In the case of violation of the assumptions (e.g. non-normality), a bootstrap-type or permutation test was used. Between-group statistical differences for the intakes of vitamins and minerals were analyzed using median regression models (least absolute value). Differences of proportions using dietary supplements between the groups were tested by chi-square test. The normality of variables was evaluated using the Shapiro–Wilk test. Statistical analyses were performed using Stata statistical software.

This study was conducted according to the guidelines laid down in the Declaration of Helsinki and all procedures involving human subjects were approved by the Ethics Committee of the Department of Obstetrics and Gynecology of Helsinki and Uusimaa Hospital District. Written informed consent was obtained from all subjects.

## Results

### Nutrient intake of pregnant women

No differences were apparent in the background characteristics between the subjects (*n=*234) and those who did not provide dietary intake data (*n=*47) (results not shown). Demographic characteristics of the pregnant subjects are presented in Tables 1 and 2. Nulliparous women were younger and more often employed than the other pregnant women. The pregnant women with a history of GDM (H-GDM) had lower BMI than the other pregnant women.

**Table 1:** Age and BMI of the participating pregnant women, with *p*-values for the differences between subgroups

			Pregnant subgroups						
				
	All (*n=*234)		NP (*n=*104)		NH-GDM (*n=*58)		H-GDM (*n=*72)		*p*
Age (years) mean, SD	32	5	30	5	33	5	33	4	<0.001
BMI (kg/m^2^) mean, SD	32	5.5	34	3.4	34	3.7	26	5	<0.001

NP, nulliparous; NH-GDM, with no history of gestational diabetes; H-GDM, with a history of gestational diabetes.

**Table 2:** Demographic characteristics of the participating pregnant women, with *p*-values for the differences between subgroups

			Pregnant subgroups						
				
	All (*n=*234)		NP (*n=*104)		NH-GDM (*n=*58)		H-GDM (*n=*72)		
					
	*n*	%	*n*	%	*n*	%	*n*	%	*p*
Highest education^a^									0.06
No professional education	21	9	10	9	6	10	5	7	
Vocational course or school	70	30	27	26	26	45	17	24	
Vocational diploma/degree	70	30	33	32	17	29	20	28	
Academic degree	72	31	33	32	9	16	30	42	
Employment status									<0.001
Employed	168	72	93	89	31	53	44	61	
Housewife or maternity leave	49	21	0	0	24	41	25	35	
Other	17	7	11	11	3	5	3	4	
Married or cohabiting	225	96	100	96	54	93	71	99	0.27
Non-smokers	225	96	100	96	54	93	71	99	0.27
BMI (kg/m^2^) categories									<0.001
<25	39	17	0	0	0	0	39	54	
25–29.9	18	8	0	0	0	0	16	22	
30–34.9	120	51	68	65	40	69	14	19	
≥35	57	24	36	35	18	31	3	4	

a One subject had missing data on highest education; NP, nulliparous; NH-GDM, with no history of gestational diabetes; H-GDM, with a history of gestational diabetes.

The mean intakes of carbohydrates, SFA, EFA, and dietary fiber were below the Finnish national nutrition recommendations (Table 3). Pregnant women with no history of GDM (NH-GDM) had lower intake of protein, whereas H-GDMs had lower intake of sucrose than the other pregnant subgroups with and without adjustment for age and BMI. The median intakes of folate, vitamin D, vitamin A, and iron from food sources among pregnant women were below the recommendation (Table 4). H-GDMs had higher intakes of vitamin D and iodine from food sources than the other pregnant women but no statistical difference occurred after adjustment for age and BMI. Median total intakes of vitamins and minerals, excluding vitamin A, were above the recommended lower limit of intake (Table 5). No differences were found in total intakes of vitamins and minerals between the pregnant subgroups, with or without adjustments for age and BMI. The prevalence of dietary supplement use among pregnant women was 77%, and the most commonly used supplements were vitamin D, folic acid, vitamin E, and vitamin C (Table 6). No differences in supplement use were found between pregnant subgroups.

**Table 3:** Macronutrient intake of pregnant Finnish women at high risk of gestational diabetes and *p*-values for the differences between subgroups

				Pregnant subgroups								
							
		All (*n=*234)		NP (*n=*104)		NH-GDM (*n=*58)		H-GDM (*n=*72)				
								
		Mean	SD	Mean	SD	Mean	SD	Mean	SD	Crude *p*	Adjusted *p* a	Rec^b^
Energy	kJ	7,967	1,861	7,829	1,931	8,010	1,921	8,031	1,670	0.82	0.59	
	kcal	1,903	444	1,883	463	1,925	471	1,915	397			
Protein	E%	18	3	18	3	17	3	18	3	0.002	0.002	10–20
Carbohydrates	E%	46	6	46	6	46	6	45	5	0.28	0.65	50–60
Total FA:s	E%	33	6	33	6	34	6	33	5	0.34	0.39	25–35
SFA	E%	12	3	12	3	13	3	12	3	0.42	0.29	10
MUFA	E%	11	2	11	2	11	2	11	2	0.61	0.78	10–15
PUFA	E%	6	1	6	1	6	1	6	1	0.19	0.41	5–10
EFA	E%	4.5	1.3	4.5	2	4.6	1	4.6	1	0.78	0.77	5
ω-3 PUFA	E%	2	0	2	0	2	0	2	0	0.31	0.20	1
Sucrose	E%	9	4	10	4	10	4	7	3	<0.001	<0.001	<10
Dietary fiber	g/MJ	2.9	0.9	2.8	1	2.9	1	3.1	1	0.07	0.30	3

a Adjusted for BMI and age.

b Rec, Finnish nutrition recommendations 2005 (19). FA, fatty acid; SFA, saturated fatty acid; MUFA, monounsaturated fatty acid; PUFA, polyunsaturated fatty acid; EFA, essential fatty acids (linoleic acid and alfa-linolenic acid); NP, nulliparous; NH-GDM, with no history of GDM; H-GDM, with a history of GDM.

**Table 4:** Intake of vitamins and minerals from food in pregnant Finnish women and *p*-values for the differences between subgroups

				Pregnant subgroups								
							
		All (*n=*234)		NP (*n=*104)		NH-GDM (*n=*58)		H-GDM (*n=*72)				
								
		Median	IQR	Median	IQR	Median	IQR	Median	IQR	Crude *p*	Adjusted *p* a	Rec^b^
Vitamins:												
C	mg	130	91, 181	150	101, 191	117	78, 173	127	91, 165			85
	mg/MJ	16	12, 24	18	14, 25	15	9, 22	17	11, 23	0.26	0.30	
E	mg	10	8, 13	10	7, 13	11	8, 13	11	9, 13			10
	mg/MJ	1.3	1.1, 1.5	1.3	1.1, 1.5	1.3	1.2, 1.5	1.3	1.2, 1.5	0.93	0.71	
A	µg	679	488, 884	640	473, 869	621	441, 884	721	553, 896			800
	µg/MJ	82	65, 110	79	65, 112	77	59, 100	86	75, 112	0.40	0.69	
D	µg	6	4, 9	6	4, 8	6	4, 9	8	5, 11			10
	µg/MJ	0.8	0.5, 1.2	0.7	0.5, 1.1	0.7	0.5, 1.1	0.9	0.7, 1.5	0.006	0.20	
Folate	µg	272	224, 326	269	224, 313	259	198, 318	298	238, 340			400
	µg/MJ	35	30, 41	35	30, 41	33	28, 40	38	32, 42	0.08	0.55	
Calcium	mg	1,146	879, 1,410	1,138	867, 1,383	1,064	856, 1,319	1,195	976, 1,472			900
	mg/MJ	145	116, 178	144	115, 182	138	112, 156	151	124, 186	0.42	0.67	
Iron	mg	12	10, 14	12	9, 13	11	10, 13	12	11, 15			
	mg/MJ	1.5	1.3, 1.7	1.5	1.3, 1.7	1.5	1.3, 1.6	1.6	1.4, 1.8	0.15	0.73	
Iodine	µg	235	193, 288	233	194, 285	213	180, 256	261	212, 306			175
	µg/MJ	31	25, 35	31	25, 35	28	23, 32	32	38, 36	0.02	0.11	

a Adjusted for BMI and age.

b Rec, Finnish national nutrition recommendations 2005 (19). IQR, interquartile range; NP, nulliparous; NH-GDM, with no history of GDM; H-GDM, with a history of GDM.

**Table 5:** Total intake of vitamins and minerals in pregnant Finnish women and *p*-values for the differences between subgroups

				Pregnant subgroups								
							
		All (*n=*234)		NP (*n=*104)		NH-GDM (*n=*58)		H-GDM (*n=*72)				
								
		Median	IQR	Median	IQR	Median	IQR	Median	IQR	Crude *p*	Adjusted *p* a	Rec^b^
Vitamins:												

C	mg	178	124, 248	198	139, 255	164	109, 235	162	114, 245			85
	mg/MJ	23	15, 32	25	19, 33	20	13, 33	21	15, 31	0.08	0.08	
E	mg	15	11, 21	15	10, 21	17	11, 21	15	11, 21			10
	mg/MJ	1.9	1.3, 2.7	1.9	0.4, 2.7	2.0	1.4, 2.6	1.8	1.3, 2.7	0.45	0.23	
A	µg	691	490, 897	652	473, 876	680	454, 912	726	580, 901			800
	µg/MJ	83	65, 111	79	65, 114	79	59, 103	87	75, 114	0.29	0.82	
D	µg	12	8, 17	12	8, 16	13	9, 19	12	8, 18			10
	µg/MJ	1.6	1.1, 2.2	1.5	1.0, 2.2	1.6	1.2, 2.4	1.6	1.0, 2.3	0.90	0.97	
Folic acid	µg	495	299, 671	488	292, 654	493	306, 677	524	303, 680			400
	µg/MJ	63	37, 85	63	36, 85	61	36, 91	63	40, 80	0.98	0.82	
Calcium	mg	1,226	977, 1,505	1,247	923, 1,496	1,174	897, 1,441	1,229	1,040, 1,531			900
	mg/MJ	152	126, 195	152	129, 198	148	124, 176	157	127, 192	0.84	0.27	
Iron	mg	15	11, 28	16	11, 29	14	10, 33	15	11, 23			
	mg/MJ	1.8	1.4, 3.5	1.9	1.5, 3.7	1.7	1.4, 3.6	1.8	1.5, 3.0	0.73	0.81	
Iodine	µg	287	224, 366	286	213, 388	278	211, 356	294	251, 363			175
	µg/MJ	36	29, 47	36	29, 49	36	27, 46	36	31, 45	0.93	0.72	

a Adjusted for BMI and age.

b Rec, Finnish national nutrition recommendations 2005 (19). NP, nulliparous; NH-GDM, with no history of GDM; H-GDM, with a history of GDM; IQR, interquartile range.

**Table 6:** The number and proportion of supplement users among pregnant Finnish women and *p*-values for the differences between the above-mentioned subgroups

			Pregnant subgroups						
				
	All (*n=*235)		NP (*n=*104)		NH-GDM (*n=*58)		H-GDM (*n=*72)		
					
	*n*	%	*n*	%	*n*	%	*n*	%	*p* a
Any supplement	181	77	81	78	48	83	52	72	0.36
Vitamins:									
C	128	55	57	55	35	60	36	50	0.50
E	133	57	57	55	38	66	38	53	0.30
A	7	3	2	2	2	3	3	4	
D	169	72	77	74	45	78	47	65	0.25
Folic acid	149	64	63	61	40	69	46	64	0.57
Calcium	45	19	24	23	11	19	10	14	0.31
Iron	96	41	46	44	28	48	22	31	0.08
Iodine	106	45	47	45	28	48	31	43	0.84
DHA	21	9	8	8	6	10	7	10	0.82
EPA	23	10	9	9	7	12	7	10	0.78

a The chi-square statistic is significant at the 0.05 level. DHA, docosahexaenoic acid; EPA, eicosapentaenoic acid; NP, nulliparous; NH-GDM, with no history of GDM; H-GDM, with a history of GDM.

## Discussion

### Main results

In the present study, the pregnant women at elevated risk of GDM had high intake of fat, intake of SFA higher than recommended, and intake of carbohydrate lower than recommended. H-GDMs had a lower proportion of total energy supplied by sucrose than NH-GDMs. Intakes of vitamins D and A, folate, and iron from food sources were below the Finnish nutrition recommendations, but total intakes, excluding vitamin A, were above the recommended lower level. Most pregnant women used dietary supplements, mainly vitamin D, folic acid, and vitamins E and C.

### Limitations

It is possible that more health conscious and educated women participated in RADIEL study. The background characteristics of those RADIEL participants who provided dietary data and those who did not, however, were similar. To minimize the bias of differing background characteristics during assessment of the between-group differences in nutrient intakes, we corrected the model by adjusting for BMI and age. Misreporting unhealthy eating patterns is common among obese and pregnant women (20, 21). Up to 45% of pregnant women may underreport their food intake.

To assess the adequacy of nutrient intake, a comparison with average nutrient requirements would fit better than the recommended daily intake (22). The Nordic nutrition recommendations, however, lack average nutrient requirements for pregnant women (16). Comparison with the recommendations should thus be cautious because it may result in overestimation of the inadequate intake.

### Interpretation

Among the pregnant women, carbohydrate intake was lower and fat intake higher than in previous Finnish studies among pregnant women at high risk of GDM (23, 24). These differences probably reflect a recent change in carbohydrate proportions in the diets of the Finnish population (25).

The proportions of energy-yielding nutrients in the diet of pregnant women may not be optimal for preventing GDM. Intakes low in carbohydrate, high in total fat, and high in SFA are associated with increased risk of GDM (13, 26). In H-GDMs, the sucrose intake was low, which may be beneficial in preventing GDM (12). The lower intake of sucrose in this group compared to the other pregnant women was likely a result of dietary counseling during the prior GDM-affected pregnancy. A difference in macronutrient intakes occurring only in the intake of sucrose and protein demonstrates the need for a proper dietary intervention for women at high risk of GDM. Dietary counseling for these women should emphasize the quality of fats and sufficient intake of dietary fiber.

The pregnant women had sufficient total intake of vitamin D, which is a new finding in Finland. The difference between this finding and previous studies is probably due to increased awareness. Vitamin D supplement use among pregnant women has increased during the past decade (27), as has vitamin D fortification of Finnish dairy products.

The pregnant women had sufficient intake of folic acid compared to the recommendation. This finding is also different compared to previous studies, where intake of folic acid has consistently fallen below the recommendations (23, 28–30). The intake falls slightly below the new recommendation, announced in 2014, of 500 µg/day, however, and thus maternity clinics should continue to emphasize the importance of sufficient folic acid intake. Optimal intake of folate in early pregnancy is crucial for preventing neural tube defects in the fetus (31).

As in previous studies, supplement use among pregnant women was high (27, 29), the most commonly used nutrients being vitamin D, folic acid, and vitamins E and C. Supplementation with vitamin D and folic acid was justified due to insufficient supply from food sources. However, supplementation with vitamins C and E and calcium was mostly unnecessary and probably resulted from the large number of multivitamin supplements targeted at pregnant women on the market in Finland.

Assessing the intake of vitamin A may require a food recording period longer than 3 days (32). However, the pregnant women in our study seemed to completely avoid foods with high vitamin A content, especially liver products, which could be partly because of the possible teratogenic effect of high vitamin A intake (33). Previous studies with adequate intakes of vitamin A among pregnant women (29, 30) may reflect constricted awareness of its teratogenicity back then. Our study suggests that some pregnant women at high risk of GDM may have insufficient intake of vitamin A. Dietary sources of vitamin A, other than liver products, should be recommended during pregnancy, namely, meat, eggs, and vegetables.

These results add to the scarce knowledge about the nutrient intake of pregnant women at high risk of GDM due to obesity or to a history of GDM. To our knowledge, this is the first study to report the effect of a history of GDM on the nutrient intake of the woman in a future pregnancy.

## Conclusions

Pregnant women at high risk of GDM have excessive SFA in their diet, which may further increase their risk of GDM. A history of GDM seems to modify sucrose intake in a beneficial direction during future pregnancy. Except for vitamin A, pregnant women have micronutrient intake at the recommended level when the diet is supplemented with vitamin D and folic acid. Those pregnant women who do not take vitamin D and folic acid supplements should be identified, monitored, and given dietary advice. The subjects of the current study were rather well educated yet still maintained inadequate nutrient intake, which highlights the need for dietary intervention for women at elevated risk of GDM. Whether dietary intervention can improve the diet of these women and thus prevent the onset of GDM is an object for future studies. Because of low intake, attention to sufficient vitamin A intake may be needed in dietary counseling at the maternity clinics.
